# Quantitative analysis of proteins of metabolism by reverse phase protein microarrays identifies potential biomarkers of rare neuromuscular diseases

**DOI:** 10.1186/s12967-015-0424-1

**Published:** 2015-02-18

**Authors:** Fulvio Santacatterina, Margarita Chamorro, Cristina Núñez de Arenas, Carmen Navarro, Miguel A Martín, José M Cuezva, María Sánchez-Aragó

**Affiliations:** Departamento de Biología Molecular, Centro de Biología Molecular, c/ Nicolás Cabrera 1, Universidad Autónoma de Madrid, 28049 Madrid, Spain; Centro de Investigación Biomédica en Red de Enfermedades Raras (CIBERER), Madrid, Spain; Instituto de Investigación Hospital 12 de Octubre, ISCIII, Madrid, Spain; Instituto de Investigación Biomédico de Vigo (IBIV), Hospital Universitario de Vigo, Meixoeiro, 36200 Vigo, Spain; Laboratorio de Enfermedades Mitocondriales y Neuromusculares, Hospital Universitario 12 de Octubre, 28041 Madrid, Spain

**Keywords:** Energy metabolism, Mitochondria, Biomarkers, Neuromuscular diseases, Rare diseases

## Abstract

**Background:**

Muscle diseases have been associated with changes in the expression of proteins involved in energy metabolism. To this aim we have developed a number of monoclonal antibodies against proteins of energy metabolism.

**Methods:**

Herein, we have used Reverse Phase Protein Microarrays (RPMA), a high throughput technique, to investigate quantitative changes in protein expression with the aim of identifying potential biomarkers in rare neuromuscular diseases. A cohort of 73 muscle biopsies that included samples from patients diagnosed of Duchenne (DMD), Becker (BMD), symptomatic forms of DMD and BMD in female carriers (Xp21 Carriers), Limb Girdle Muscular Dystrophy Type 2C (LGMD2C), neuronal ceroid lipofuscinosis (NCL), glycogenosis type V (Mc Ardle disease), isolated mitochondrial complex I deficiency, intensive care unit myopathy and control donors were investigated. The nineteen proteins of energy metabolism studied included members of the mitochondrial oxidation of pyruvate, the tricarboxylic acid cycle, β-oxidation of fatty acids, electron transport and oxidative phosphorylation, glycogen metabolism, glycolysis and oxidative stress using highly specific antibodies.

**Results:**

The results indicate that the phenotype of energy metabolism offers potential biomarkers that could be implemented to refine the understanding of the biological principles of rare diseases and, eventually, the management of these patients.

**Conclusions:**

We suggest that some biomarkers of energy metabolism could be translated into the clinics to contribute to the improvement of the clinical handling of patients affected by rare diseases.

**Electronic supplementary material:**

The online version of this article (doi:10.1186/s12967-015-0424-1) contains supplementary material, which is available to authorized users.

## Background

Genetic alterations that result in cellular dysfunction are usually accompanied by changes in the expression of proteins of energy metabolism. A good example in this regard is provided by the chromosomal abnormalities and multiple genetic mutations that promote cancer and that converge in the reprogramming of energy metabolism [1,2]. In fact, energy metabolism provides a valuable tool as biomarker of disease progression [3,4] and of the eventual response to therapy [5,6]. Most rare diseases have no cure and the living with the disease until the patient becomes diagnose, if at all, is a heavy burden for the patient and for their families. Hence, there is an urgent need to identify biomarkers that could aid in the diagnosis and/or follow up of these patients in order to increase the understanding of disease processes that could be further translated into potential therapeutic interventions.

Reverse Phase Protein Microarrays (RPMA) is a high-throughput technique that allows the quantification of a given protein in minute amounts of sample from biological specimens [7,8]. The application of this technology allows the identification of new markers of diagnosis, the establishment of correlations between protein markers and the severity and progression of the disease and, eventually, of the response to a given treatment [7,8]. However, a bottle-neck in the application and development of RPMA is the availability of high-affinity and specific monoclonal antibodies that could be used in the unambiguous characterization of a particular phenotype, either because the antibodies have not been developed or they lack the required specificity [9,10]. As part of an ambitious project aimed at translating the “signature” of energy metabolism to bed-side application, we are producing specific monoclonal antibodies against enzymes of glycolysis and mitochondrial oxidative phosphorylation [11-13] to be applied in RPMA or any other technique, with the aim of facilitating patient management affected with different pathologies [4].

In the present investigation, we have produced additional antibodies against proteins of energy metabolism and applied RPMA technology to quantify and study the putative relevance of nineteen of these proteins as potential biomarkers in a cohort of seventy three muscle biopsies including control donors and patients affected of rare neuromuscular diseases such as Duchenne (DMD) and Becker (BMD) dystrophies, symptomatic forms of DMD and BMD in female carriers (Xp21 Carriers), Limb Girdle Muscular Dystrophy Type 2C (LGMD2C), neuronal ceroid lipofuscinosis (NCL), glycogen storage disease type V (Mc Ardle disease), isolated deficiency of mitochondrial respiratory chain complex I and intensive care unit myopathy. The results obtained indicate that enzymes of energy metabolism might offer relevant biomarkers that could aid the understanding of the biology of rare neuromuscular diseases and, eventually, the management of these patients.

## Methods

### Patients and protein extraction

A cohort of deltoid and quadriceps muscle biopsies of control donors (n = 20) and patients affected of neuromuscular diseases including Duchenne (DMD, n = 6), Becker (BMD, n = 6), symptomatic forms of DMD and BMD in female carriers (Xp21 Carriers, n = 4), Limb Girdle Muscular Dystrophy Type 2C (LGMD2C, n = 6), glycogenosis type V (McArdle disease, n = 7), deficit of mitochondrial Complex I (n = 12), neuronal ceroid lipofuscinosis (NCL, n = 6) and intensive care unit myopathy (n = 6) were processed. Frozen tissue sections obtained from surgical specimens were provided from Instituto de Investigación Biomédica de Vigo, Vigo and from Instituto de Investigación Hospital 12 de Octubre, Madrid, Spain. Routine histopathological study and appropriate molecular and clinical diagnosis of all the cases studied had been previously performed. The samples were obtained with informed consent following the Declaration of Helsinki and coded for anonymity to protect patient confidentiality. The Institutional Review Board approved the project. For protein extraction, the samples were homogenized in T-PER Tissue Protein Extraction Reagent (ThermoScientific, Inc. Madrid, Spain) containing protease inhibitors (protease Cocktail Tablets; Life Sciences, Madrid, Spain) in a 1:5 (w/v) ratio, and further freeze–thawed three times in liquid nitrogen [4]. The protein concentration was determined with the Bradford reagent (Bio-Rad, Inc. Madrid, Spain) using BSA as standard.

### Cloning strategies, protein expression and purification

To obtain the recombinant proteins to be used for antibody production the cDNAs encoding human lactate dehydrogenase A (LDH-A; NP_005566), NADH-ubiquinone oxidoreductase α-subunit 9 (NADH-sub9; NM_005002), aconitase I (ACO1; NM_002197), glycerol-3-phosphate dehydrogenase 1 (GPD1; NM_005276) and citrate synthase (CS; NM_004077) were amplified by polymerase chain reaction as previously described [11]. The sequences of the forward (F) and reverse (R) primers used were as follows: LDH-A (F: 5′-GAGCTCATGGCAACTCTAAAGGATCAGC-3′; R: 5′- GCGGCCGCAAATTGCAGCTCCTTTTGGAT - 3′); NADH-sub9 (F: 5′-CGGGAGCTCATGGCGGCTGCCG-3′; R: 5′-ATAGTTTAGCGGCCGCTGAATGTTGACGGTCTTG-3′); ACO1 (F: 5′-GAGCTCATGCGTGTCATCCTGCAGGACTTT - 3′; R: 5′ GCGGCCGCGATGGTTCCAGCAATTGCAT - 3′); GPD1 (F: 5′ CGGGAGCTCATGGCTAGCAAGAAAGTCT - 3′; R: 5′- ATAGTTTAGCGGCCGCCACATATGTTCTGGATGATT - 3′); and CS (F: 5’ CGGAAGCTTATGGCTTTACTTACTGCG-3’; R: 5’-ATAGTTTACACGTGACCCACCCTGACTTAGA-3’). Amplicons were first cloned into pGEM-T easy vector (Promega, Madison, WI) and after into pQE-Trisystem (for details see [11]). The resulting plasmids, pQE-LDH-A, pQE-NADH-sub9, pQE-ACO1, pQE-GPD1 and pQE-CS were used to transform Escherichia coli M15/pREP4 cells. It should be noted that pQE-ACO1 expresses a truncated version of ACO1. After induction of protein expression by adding IPTG (1 mM), the cells were resuspended in buffer A containing 100 mM NaH_2_PO_4_, 300 mM NaCl, pH 8.0 supplemented with lysozyme 1 mg/ml. The expressed proteins were purified using either Strep-Tactin or metal ion affinity chromatography Ni-NTA superflow resins (Qiagen, Hilden, Germany). The purity of the proteins was estimated by fractionation on SDS-PAGE (Additional file 1: Figure S1).

### Antibody production

BALB/c mice were immunized by intraperitoneal injection with various dosages of the purified proteins (20 μg). Serum was obtained from mice and tested for reactivity against the recombinant and native proteins by western blotting (Additional file 1: Figure S1). When a titer higher than 1000 was attained, hybridomas were produced by fusing spleen cells with myeloma SP2 or NS-1 cells with polyethylene glycol in HAT-RPMI 1640 medium according to standard hybridoma techniques [11-13]. Supernatants of the hybridomas were screened by indirect ELISA on polystyrene plates coated with the recombinant proteins (0–150 ng per well). Bound antibodies were detected using horseradish peroxidase–labeled goat antimouse antibodies (1:1000) (DAKO, Carpinteria, CA). After the final washing, 100 μl of OPD solution (Sigma, St. Louis, MO) was added, and the color reaction was developed for 15 minutes and stopped by the addition of 18 M H_2_SO_4_. Optical density at 490 nm was determined in a FluoStar Optima (BMG Labtech, Offenburg, Germany) apparatus. The positive colonies were cloned by limiting dilution. Mouse monoclonal antibodies were purified with Montage antibody purification kit (Millipore, Billerica, MA) according to the supplier’s instructions. Highly specific monoclonal antibodies against NADHs9 15/22-5, Aco-I 13/18-1, GPD1 P5A1-1 and LDHA 4D3-A1 were obtained (Additional file 1: Figure S1). Specific polyclonal mouse antibodies against citrate synthase were used in the study (Additional file 1: Figure S1) because we failed in obtaining reliable hybridomas for this protein.

### Printing and processing of reverse phase protein microarrays

Samples from patient biopsies were diluted in PBS (137 mM NaCl, 2.7 mM KCl, 10 mM Na2HPO4 and 1.8 mM KH2PO4 pH 7.4) to a final protein concentration of 1 μg/μl before printing. Serially diluted protein extracts (0–1 μg/μl) derived from HCT116 colocarcinoma cells were also prepared to asses printing quality and the linear response of protein recognition by the antibodies used. A solution of BSA (1 μg/μl) was also prepared for printing as internal negative control. Approximately, 1 nl volume of each sample was spotted in quadruplicate onto nitrocellulose-coated glass slides (FAST Slides, Schleicher & Schuell BioScience, Inc. Dassel, Germany) using a BioOdyssey Calligrapher MiniArrayer printer (Bio-Rad Laboratories, Inc., Madrid, Spain) equipped with a solid pin (MCP310S) at constant humidity of 45% and 10°C and 16°C for the plate and chamber, respectively. After printing, arrays were allowed to dry at room temperature for 16 hours and further blocked in PBS-T containing 5% skimmed milk. After, the arrays were incubated overnight at 4°C with the indicated concentrations of the following highly specific primary monoclonal antibodies (mAbs): anti-β-F1-ATPase (1:150), anti-Hsp60 (1:150), anti-GAPDH (1:250) and anti-PK (1:150) from [4], anti-IF1 (1:50) from [12], anti-α-F1-ATPase (1:250), anti-COXI (1:85) and anti-COXIV (1:50) from Molecular Probes (Madrid, Spain), anti-PDH (1:50) and anti-SDH (1:50) from Invitrogen (Madrid, Spain), anti-SOD2 (1:100), anti-PYGM (1:200), anti-β-actin (1:1000) from Sigma (Madrid, Spain), and the additional home-made anti-NADHs9 (1:1000), anti-LDH-A (1:2,500), anti-ACO1 (1:250) and anti-GPD1 (1:1000). The polyclonal mouse anti-CS (1:500) and rabbit anti-CPT1M (1:25) from Santa Cruz (Heidelberg, Germany) and anti-HADHA (1:1000) from Abcam (Cambridge, UK) were also used. Each array was incubated with each antibody independently. After incubation the arrays were washed with PBS-T and further incubated with a donkey anti-mouse or donkey anti-rabbit secondary antibody conjugated with alexa-488 (Invitrogen, Madrid, Spain). Microarrays were scanned using a Typhoon 9410 scanner (GE Healthcare, Inc. Madrid, Spain). The mean fluorescent intensity of the spots was quantified using FIJI software (N.I.H., USA) and converted into arbitrary units of expressed protein/ng of total protein in the tissue extract using the expression obtained in the linear plot of the HCT116 cell line as standard. The technical variance of the arrays calculated by the squared coefficient of variation (SCV = σ*100/ |x|) was 8.6 ± 0.6.

### Statistical analysis

Distribution of molecular markers was studied by using a two-tailed Student's *t* test. Analysis of variance (ANOVA) with post hoc Dunnett's test used for multiple comparisons to the control and analysis of variation in samples with box plot diagrams were performed using the PASW statistics 18 software package. For the expression profiles of metabolic markers data were reformatted by calculating the log(2) of the expression level in each sample relative to the mean expression level in normal samples. We used the Cluster Program from “Expression Profiler Clustering home page” at http://ep.ebi.ac.uk/EP/EPCLUST using the Euclidean distances and average linkage method (Weighted Group Average, WPGMA). The results shown are means ± S.E.M. A p < 0.05 was considered statistically significant.

## Results

### Validation of the antibodies used for RPMA

**Figure 1:**
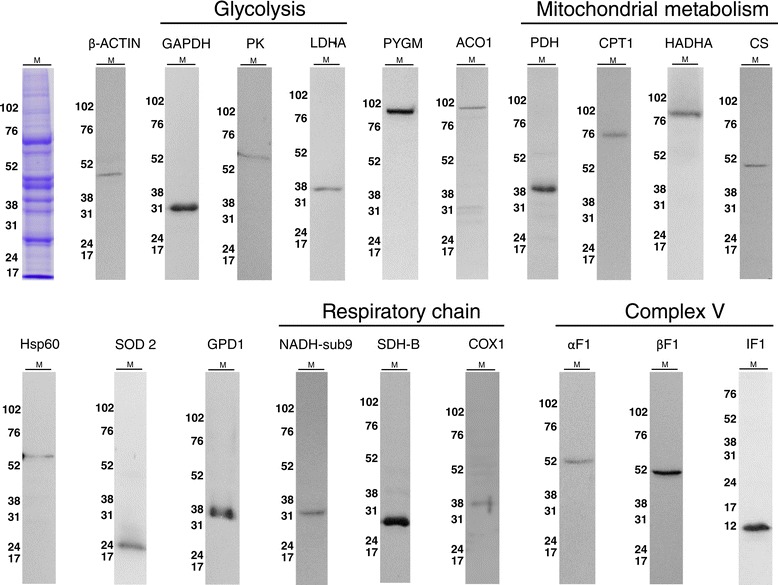
**Validation of the antibodies used for application in RPMA.** 30–40 μg of protein derived from human muscle (M) were fractionated on SDS-PAGE gels (see Coomasie blue stained track on top-left), blotted against the indicated antibodies and processed for western blotting. Only antibodies that recognize a single protein band of the expected molecular mass were used in the study. The migration of molecular mass markers is indicated to the left.

High affinity and specific monoclonal antibodies against proteins of energy metabolism are the rate-limiting tools required for the successful application of RPMA technology [14]. The metabolic pathways interrogated in this study included the degradation of glycogen (PYGM), glycolysis (GAPDH, PK, LDHA), the shuttling of cytosolic electrons to mitochondria (GPD1), mitochondrial decarboxylation of pyruvate (PDH), the mitochondrial import and oxidation of fatty acids (CPT1, HADHA), the Krebs cycle (CS), the electron transport chain (NADHs9, SDHB, COX1), the ATP synthase as engine of oxidative phosphorylation (αF1, βF1, IF1), cytosolic (ACO1) and mitochondrial (SOD2) markers of oxidative stress. In addition, cellular (β-actin) and mitochondrial (Hsp60) structural markers were included to normalize changes in protein expression. The selection of target proteins was mostly based on the facts that they are abundant proteins in core pathways of energy provision. Hence, a first step of this study was to validate the specificity of the antibodies to be used in RPMA by western blotting using human muscle extracts (Figure 1). Both the antibodies commercially available or made in the lab were tested [11,12] (and see Additional file 1: Figure S1). The antibodies used in this study recognized one single protein band of the expected molecular weight in human muscle samples (Figure 1), validating their utilization for the purpose of quantification protein expression in RPMA techniques.

### Protein expression in human muscle biopsies

**Figure 2:**
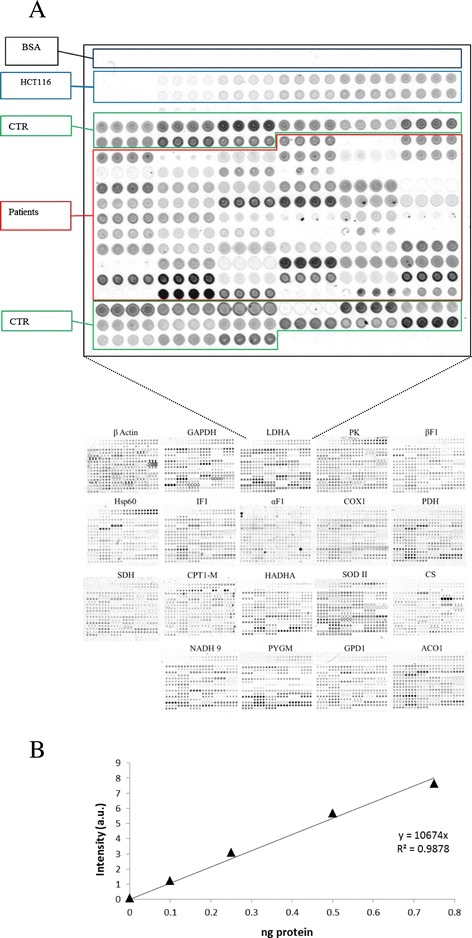
**Printing of RPMA. A**, Scheme of RPMA printing processed for anti-LDH-A is shown magnified. One nl samples were spotted in quadruplicate. Black boxed: negative controls of BSA; Blue boxed: standard curves of HCT116 cells; Green boxed: tissue samples from control donors; Red boxed: tissue samples from patients. Below are shown representative RPMAs processed with other antibodies. **B**, The plot illustrates the linear correlation that exists between the fluorescence intensity (arbitrary units, a.u.) and the amount of LDH-A protein in HCT116 cell lysates. Protein concentrations in the biopsies were calculated according to the fluorescence intensity obtained in the linear plot of HCT116 cells. For other details see Additional file 2: Figure S2.

A representative protein microarray illustrating the printing protocol of human muscle biopsies developed with antibodies against the glycolytic LDH-A is shown in Figure 2A. Arrays developed with other antibodies are shown below (Figure 2A). Protein extracts from muscle biopsies of control (green boxed in Figure 2A) and different neuromuscular diseases (red boxed in Figure 2A) were prepared and spotted onto RPMA in quadruplicate from left to right (Figure 2A). Increasing amounts of BSA (black boxed in Figure 2A) were spotted in the array as a control of the background of the assay. The arrays also contained increasing protein amounts of cellular extracts derived from HCT116 cells (blue boxed in Figure 2A). The HCT116 extracts revealed a linear increase in fluorescent intensity as the amount of protein increased in the spot (Figure 2B), providing the standard curve of the assay (see also Additional file 2: Figure S2). The arrays illustrated the specific recognition of the corresponding antigen in minute amounts of printed protein of HCT116 extracts as well as in the biopsies (Figure 2A). As expected, no fluorescent signal was observed in BSA containing spots (Figure 2A), which provides the background of the technique by non-specific absorption of labeled antibodies to the proteins spotted. The quantification of the expression of each marker in control (n =20) and patient (n =53) biopsies was calculated by interpolating the fluorescent intensity signal obtained in the sample in the linear plot of HCT116 cells (Figure 2A and Additional file 2: Figure S2) and expressed as fold of control. Array duplicates show that the results obtained are highly reproducible validating the robustness of the technique for quantitative purposes (see Additional file 3: Figure S3).

The analysis of protein expression comparing patient’s samples to their respective controls taking into consideration the muscle type of origin (deltoid or quadriceps; see printing scheme of the arrays on Figure 2) provided no significant differences. Hence, and for the sake of simplicity, we decided to present the data of controls all together (Table 1). The results in Table 1 and Additional file 4: Figure S4 summarizes the expression of nineteen proteins involved in different cellular activities and of the ratios derived from them, in different myopathies when compared to controls. The box plot in Additional file 4: Figure S4 illustrate the individual variation of the expression of the markers in each group of the pathologies studied as well as the outliers. In general, only Complex I deficient patients and patients affected of Duchenne muscular dystrophy showed a significant increase in several mitochondrial markers involved in energy metabolism when compared to controls (Table 1 and Additional file 4: Figure S4). Paradoxically, NADH-dehydrogenase subunit 9 expression was significantly increased in Complex I deficient patients (Table 1 and Additional file 4: Figure S4). A significant reduction in SOD2 expression was also noted in Duchenne and Becker muscular dystrophies (Table 1 and Additional file 4: Figure S4). Similarly, expression of the inhibitor of the mitochondrial H^+^-ATP synthase, IF1, was also significantly diminished in patients affected of intense care unit and NCL myopathies (Table 1 and Additional file 4: Figure S4). This finding is consistent with the recent observation that inhibition of IF1 ameliorates severe mitochondrial respiratory chain dysfunctions [15]. In general, mitochondrial markers showed slight variations when compared to controls in glycogenosis type V, Becker and symptomatic forms of DMD and BMD in female carrier muscular dystrophies, patients affected of intensive care unit myopathy and NCL (Table 1 and Additional file 4: Figure S4).

**Table 1:** **Relative expression of proteins of energy metabolism in muscle biopsies of neuromuscular diseases**

		**Rare Diseases**							
		**Metabolic**		**Muscular**	**Dystrophies**			**Neurodegenerative**	**Intesive**-**Care Unit myopathy**
	**Controls**	**Mitochondrial**	**Glycogenosis**	**DMD**	**BMD**	**C. Xp21**	**LGMD 2C**	**NCL**	**ICU**
		**Complex I**	**type V**						
**Mitochondrial Markers**									
PDH E1α	1.0 ± 0.1	1.3 ± 0.1	0.6 ± 0.1	1.3 ± 0.2	1.3 ± 0.4	0.7 ± 0.1	0.8 ± 0.1	0.7 ± 0.1	0.6 ± 0.1
SDH	1.0 ± 0.1	1.4 ± 0.1 **	0.9 ± 0.1	1.4 ± 0.2 *	1.1 ± 0.2	0.9 ± 0.2	0.6 ± 0.1 *	1.0 ± 0.2	0.8 ± 0.1
CS	1.0 ± 0.1	1.5 ± 0.2 *	0.8 ± 0.1	1.1 ± 0.3	0.8 ± 0.1	1.4 ± 0.4	0.5 ± 0.2	1.1 ± 0.3	1.2 ± 0.1
Hsp60	1.0 ± 0.1	1.3 ± 0.1	0.8 ± 0.1	1.7 ± 0.2 **	1.2 ± 0.2	0.9 ± 0.2	1.3 ± 0.3	0.9 ± 0.2	0.8 ± 0.1
CPT1-M	1.0 ± 0.1	0.9 ± 0.1	1.0 ± 0.1	1.2 ± 0.2	0.7 ± 0.0 *	0.9 ± 0.0	0.7 ± 0.2	1.0 ± 0.1	1.0 ± 0.1
HADHA	1.0 ± 0.1	1.6 ± 0.2 *	1.1 ± 0.2	0.7 ± 0.1	0.7 ± 0.1	0.8 ± 0.2	0.8 ± 0.3	1.1 ± 0.2	1.4 ± 0.2
NADH-9	1.0 ± 0.1	1.8 ± 0.2 **	1.2 ± 0.1	0.9 ± 0.1	0.9 ± 0.2	1.0 ± 0.2	0.6 ± 0.2	1.0 ± 0.1	1.2 ± 0.2
COX-1	1.0 ± 0.1	1.2 ± 0.1	0.6 ± 0.1 *	1.5 ± 0.1 *	1.3 ± 0.3	0.9 ± 0.1	0.9 ± 0.2	0.8 ± 0.1	0.7 ± 0.1
α-F1	1.0 ± 0.1	1.1 ± 0.1	0.7 ± 0.2	1.4 ± 0.1 *	1.3 ± 0.5	0.8 ± 0.1	1.0 ± 0.2	1.1 ± 0.2	0.7 ± 0.1
β-F1	1.0 ± 0.1	1.3 ± 0.1 *	0.9 ± 0.1	1.2 ± 0.2	1.2 ± 0.1	1.2 ± 0.3	0.8 ± 0.1	0.8 ± 0.1	1.0 ± 0.1
IF1	1.0 ± 0.1	1.0 ± 0.1	0.7 ± 0.1	1.1 ± 0.2	1.1 ± 0.2	0.7 ± 0.1	0.8 ± 0.2	0.7 ± 0.1 *	0.6 ± 0.1 *
SOD2	1.0 ± 0.1	0.8 ± 0.1	1.0 ± 0.1	0.5 ± 0.1 *	0.5 ± 0.1 *	0.7 ± 0.2	0.8 ± 0.3	0.8 ± 0.1	1.0 ± 0.1
**Cytoplasmic Markers**									
β-Actin	1.0 ± 0.1	1.1 ± 0.1	1.3 ± 0.2	1.0 ± 0.1	1.1 ± 0.3	1.0 ± 0.1	0.8 ± 0.2	1.1 ± 0.1	1.2 ± 0.2
GAPDH	1.0 ± 0.1	0.6 ± 0.1 *	0.8 ± 0.1	0.4 ± 0.1 *	0.9 ± 0.3	0.6 ± 0.1	0.3 ± 0.1 *	0.6 ± 0.1	0.5 ± 0.1 *
PK	1.0 ± 0.1	1.0 ± 0.1	0.8 ± 0.1	0.8 ± 0.2	1.0 ± 0.2	0.8 ± 0.0	0.7 ± 0.1	0.6 ± 0.1 *	0.7 ± 0.1 *
LDH-A	1.0 ± 0.1	0.7 ± 0.1	0.6 ± 0.2	0.4 ± 0.1 *	0.6 ± 0.2	0.5 ± 0.0	0.3 ± 0.0 *	0.4 ± 0.1 *	0.4 ± 0.1 *
GPD1	1.0 ± 0.1	1.1 ± 0.1	0.9 ± 0.1	0.4 ± 0.1 **	0.7 ± 0.2	0.6 ± 0.1 *	0.6 ± 0.2 *	0.5 ± 0.1 *	0.7 ± 0.1
PYGM	1.0 ± 0.1	1.5 ± 0.2 *	0.0 ± 0.0 **	0.2 ± 0.1 **	0.7 ± 0.3	0.7 ± 0.1	0.5 ± 0.1 *	0.5 ± 0.1 *	0.4 ± 0.1 **
ACO1	1.0 ± 0.1	1.1 ± 0.1	0.9 ± 0.1	0.8 ± 0.1	1.1 ± 0.3	0.9 ± 0.2	0.7 ± 0.2	1.0 ± 0.1	0.7 ± 0.1
**Ratios**									
β-F1/GAPDH	1.0 ± 0.1	2.5 ± 0.3 **	1.1 ± 0.2	3.1 ± 0.8 **	2.2 ± 1.0 *	1.6 ± 0.4	2.9 ± 0.6 **	1.3 ± 0.3	1.7 ± 0.3 *
BEC Index	1.0 ± 0.1	2.1 ± 0.2 **	1.4 ± 0.2	1.6 ± 0.3 *	1.8 ± 0.5 *	1.9 ± 0.2 *	2.5 ± 0.5 **	1.9 ± 0.5 *	2.5 ± 0.5 **
β-F1/LDH-A	1.0 ± 0.1	2.2 ± 0.3 **	1.8 ± 0.5 *	3.5 ± 0.6 **	2.7 ± 0.8 **	1.9 ± 0.4 *	2.5 ± 0.5 **	1.8 ± 0.3 **	3.2 ± 0.7 **

The table summarizes the expression of nineteen proteins involved in different mitochondrial and cytoplasmic activities of metabolism and of the ratios derived from them in different muscle myopathies when compared to controls. Values are expressed as fold of control. The results shown are the mean values ± S.E.M. *, p < 0.05 and **, p < 0.001 when compared to controls.

However, Complex I deficient patients and patients affected of Duchenne Muscular Dystrophy, Limb Girdle Muscular Dystrophy Type 2C (LGMD2C), Neuronal Ceroid Lipofuscinosis and patients affected of intensive care unit myopathy provided significant differences in the expression of several of the cytosolic biomarkers studied when compared to control donors (Table 1 and Additional file 4: Figure S4). A significant decrease in the expression of myophosphorylase and several of the glycolytic enzymes was observed in muscular dystrophies (Duchenne Muscular Dystrophy, Limb Girdle Muscular Dystrophy Type 2C (LGMD2C), Neuronal Ceroid Lipofuscinosis and patients affected of intensive care unit myopathy) (Table 1 and Additional file 4: Figure S4). Remarkably, whereas myophosphorylase expression was increased in Complex I deficient biopsies (Table 1 and Additional file 4: Figure S4) it was completely vanished in patients affected of Glycogenosis type V (Table 1 and Additional file 4: Figure S4), consistent with the lack of myophosphorylase activity in McArdle disease [16,17]. Based on the opposite expression that exists between glycolytic and bioenergetic markers of the mitochondria during development, differentiation and in cancer [18], we calculated the bioenergetic signature of the biopsies (BEC index = βF1/Hsp60/GAPDH ratio) [3] and different alternative ratios between the catalytic subunit of the H^+^-ATP synthase (β-F1-ATPase) and the expression of GAPDH or LDH-A (Table 1 and Additional file 4: Figure S4) [19]. Remarkably, the normalized cellular content of β-F1-ATPase, as assessed by the β-F1/LDH-A ratio, was significantly augmented in all the diseases studied despite the expression of the two markers alone showed no major differences. These findings supported the β-F1-ATPase/LDH-A ratio as a bioenergetic signature of muscular affectation independent of the different genetic or epigenetics mechanisms involved in the onset of neuromuscular diseases (Table 1).

### Enzymes of metabolism as biomarkers of rare diseases

**Figure 3:**
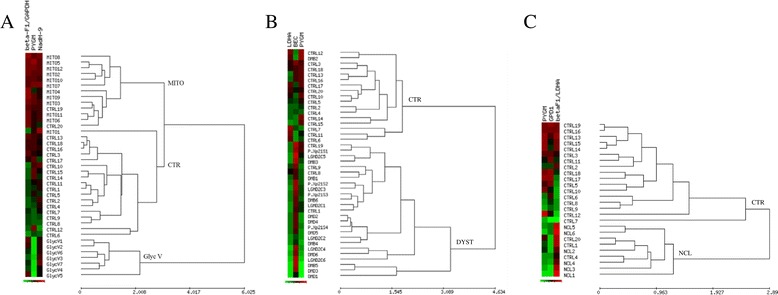
**Hierarchical clustering analyses of the biopsies using enzymes of energy metabolism.** Rows indicate type of sample, columns, proteins and derived ratios. Protein expression scores are shown normalized to the mean relative expression level in normal samples, according to a color scale (below panels): red indicates high; black, normal; and green, low expression. The dendogram (to the right of the matrix) represents overall similarities in expression profiles. The maximum and minimum values of the markers for each cluster are shown. **A**, Clustering of normal (CTR), isolated deficit of Complex I activity (MITO) and myophosphorylase (GlycV) biopsies using β-F1-ATPase/GAPDH ratio, PYGM and NADHs9 as biomarkers. **B**, Clustering of normal (CTR) and DMD, BMD, Xp21 and LGMD2C muscular dystrophies using LDH-A, BEC index and PYGM as biomarkers. **C**, Clustering of normal (CTR) and Neuronal Ceroid Lipofuscinosis (NCL) biopsies using PYGM, GPD1 and β-F1-ATPase/LDHA ratio as biomarkers.

Having observed significant differences in the expression of proteins of energy metabolism for a diverse set of rare neuromuscular diseases, we next questioned their potential as discriminatory biomarkers of disease. To this aim, we carried out unsupervised hierarchical clustering of the biopsies using the expression of 4–5 markers for aggregation purposes. This statistical method groups samples by similarity of expression in different groups or clusters [20]. To illustrate this point, the clustering of the 39 biopsies of control, deficit of Complex I activity and of the expression of myophosphorylase using the expression of NADH dehydrogenase subunit 9, myophosphorylase and the β-F1-ATPase/GAPDH ratio resulted in the distribution of the biopsies in three separate groups with a classification sensitivity of 95% and 100% for complex I and myophosphorylase deficiencies, respectively (Figure 3A). A classification specificity of 90% and 100% for controls was also observed (Figure 3A). Hierarchical clustering of 42 biopsies of control and the four muscular dystrophies studied (DMD, BMD, Xp21 and LGMD2C) using the expression of LDH-A, the BEC index and PYGM resulted in two clearly distinguished groups corresponding to controls and dystrophic patients with a classification sensitivity of 96% for the pathologic samples and a specificity of 83% for the controls (Figure 3B). The same type of analysis using 26 biopsies of control and patients affected of NCL according to the expression pattern of PYGM, GPD1 and β-F1/LDH-A ratio resulted in their distribution into two different groups, control donors and NCL patients, with a high sensitivity (100%) and specificity (85%) (Figure 3C).

Inter-individual variation of the expression of the biomarkers (Additional file 4: Figure S4) is usually a handicap for its translation because they not always qualify for clinical use. Table 2 provides a summary of the power to discriminate different neuromuscular dystrophies/myopathies by the combination of the value of the β-F1-ATPase/LDH-A ratio with the expression level of a third biomarker. It should be noted that these combinations have 100% sensitivity (Table 2) when the markers used have no outliers (Additional file 4: Figure S4) supporting their potential use in future prospective studies.

**Table 2:** **Potential diagnostic sensitivity of some metabolic biomarkers in neuromuscular dystrophies**/**myopathies**

**Disease**	**β**-**F1**/**LDH**-**A**	**AdditionalMarkers**	**Sensitivity**
DMD	(2.9-4.1)	PYGM (0.1-0.3)	100%
BMD	(1.9-3.5)	GAPDH (0.6-1.2)	86%
NCL	(1.5-2.1)	GPD1 (0.4-0.6)	100%
LGMD-2C	(2.5-3.9)	SDH (0.5-0.7)	100%

The table summarizes the power to discriminate (sensitivity) the neuromuscular dystrophies/myopathies by the combination of the value of the β-F1-ATPase/LDH-A ratio with the expression level of a third biomarker. Sensitivity was calculated according to the classification rate of true positive samples following the formula: Sensitivity = True positives/(True positives + False positives).

## Discussion

A large number of genes have been identified to be involved in different muscle-wasting neuromuscular disorders. However, knowledge of the pathophysiological mechanisms, markers of diagnosis and treatment of rare muscular diseases is scant or non-existent. Over the last two decades, remarkable progress has been made in the development of genetic-targeted therapeutic interventions for several muscular dystrophies [21]. More recently, next-generation sequencing (NGS) technologies have also been implemented for the identification of the genetic causes underlying neuromuscular diseases [22,23]. Much less studies have dealt with the analysis of the proteome of neuromuscular diseases despite having been demonstrated that the alterations in the expression of proteins of energy metabolism provides useful biomarkers in a complex genetic disorder such as cancer [5,6,19,24]. Within this context, RPMA offers a high-throughput technology for quantitative determination of the proteins that define the particular phenotype of a disease. RPMA allows the interrogation and identification of potential biomarkers, the establishment of correlations with patients’ outcome [4] and eventually, the design of future rationale therapeutic approaches based on the biomarkers identified [7,24]. In this study we have developed five additional highly specific monoclonal antibodies against proteins of energy metabolism and interrogated a cohort of muscle biopsies of patients affected of neuromuscular diseases by applying the RPMA technology using nineteen different antibodies. The purpose was the identification of proteins that could inform of the activity of energy metabolism and could provide potential biomarkers for these diseases as an additional effort to stimulate its translation to bed-side application in this orphan field of investigation. Although we have not taken into account the age of patients [25], the specific type of muscle fibers and/or the presence of necrotic areas in the biopsies, which are known factors that influence protein expression [26,27], the low dispersion of the values obtained for each marker suggests that these are not main factors contributing to the differences reported.

Muscular dystrophies are a heterogeneous group of inherited disorders characterized by progressive muscle wasting and weakness [21,28,29]. Previous studies have suggested the potential relevance of the metabolic enzymes enolase and malate dehydrogenase as biomarkers of these disorders in animal models [30]. In this regard, we have extended the list of potential metabolic biomarkers with our RPMA approach and, in agreement with findings in dystrophic dog muscle [30], we can confirm the down-regulation of the expression of most glycolytic proteins in human dystrophic muscle. In contrast to the findings in dystrophic dog muscle [30], we observed the up-regulation of mitochondrial proteins involved in oxidative phosphorylation in human dystrophic muscle. The analysis of mitochondrial function in a cardiotoxin-induced mouse model of muscular dystrophy [29] has revealed an impaired expression of mitochondrial proteins involved in the respiratory chain and oxidative phosphorylation (OXPHOS). These results contrast our findings in which it is observed, if any, a significant increase in several markers of the respiratory chain and OXPHOS (Table 1 and Additional file 4: Figure S4). These discrepancies might arise from differences between human and mouse muscle and/or from the experimental system and approaches used in these studies.

The most common and severely debilitating neuromuscular disorder, Duchenne muscular dystrophy, affects ~1 in 3,500 males and it is manifested by rapidly progressive proximal muscle wasting, respiratory insufficiency and cardiac failure that lead to premature death by the mid-20s [28]. The allelic disorder Becker muscular dystrophy is less common and milder, with relatively advanced survival age. Consistent with this, we have observed a lesser alteration of the expression of proteins of energy metabolism in biopsies of BMD patients (Table 1 and Additional file 4: Figure S4). Both diseases are caused by mutations in the dystrophin gene, one of the largest gene in the human genome, located on the X chromosome encoding a 427kD protein [28,31]. Dystrophin interacts with multiple proteins to assemble the dystrophin-associated protein complex (DAPC), a group of proteins that span the sarcolemma of the skeletal and cardiac muscle [21]. The core component of the DAPC is dystroglycan whose insufficient post-translational glycosylation is responsible for sarcoglycanopathies, a clinically heterogeneous group of congenital muscular dystrophies [28]. Within the DAPC, the subcomplex of integral proteins sarcoglycans and sarcospan provides additional mechanical support to the sarcolemma [32]. Mutations in genes encoding α, β, γ and δ subunits of the sarcoglycan complex cause sarcoglycanopathies, a subtype of recessively inherited limb-girdle muscular dystrophies (LGMDs) that also express a significant down-regulation of four cytoplasmic markers of energy metabolism, partially mimicking the findings observed in DMD (Table 1 and Additional file 4: Figure S4).

Different molecular mechanisms are responsible for the muscle wasting phenotypes [33]. However, it is noteworthy that the bioenergetic signature, as assessed by the β-F1-ATPase/LDH-A ratio, is always affected regardless of the muscular disease studied. The mechanisms promoting the global alteration of energy metabolism in the muscle of patients affected with a neuromuscular disease, best exemplified by the dramatic increase in the β-F1-ATPase/LDH-A ratio (Table 1), has remained largely unexplained. Perhaps, because it stems from the idea that only global gene expression analysis could be useful to delineate the pathophysiology of the disease. However, the situation is that genetic alterations that result in neuromuscular dystrophies vary from one specific disease to another but apparently act pleiotropically to regulate, either by genetic or epigenetic means (ICU patients), the signature of muscle energy metabolism.

Moreover, the quantification of the β-F1-ATPase/LDH-A ratio in addition to other proteins of energy metabolism provides a valuable fingerprint to discriminate between different myopathies (Table 2). Interestingly, the combination of the β-F1-ATPase/LDH-A ratio with the expression of myophosphorylase (PYGM) allows the discrimination of DMD patients with 100% of sensitivity (Table 2). Similarly, combination of this ratio with GPD1 expression discriminates patients affected of NCL with a classification sensitivity of 100% (Table 2). Moreover, the GAPDH and succinate dehydrogenase (SDH) in combination with the β-F1-ATPase/LDH-A ratio discriminate BMD (86% sensitivity) and LGMD-2C (100% sensitivity) diseases, respectively (Table 2). The metabolic markers that we have uncovered, alone or in combination with the detection of serum biomarkers [25,34,35] and/or other markers of energy metabolism that have been previously linked to muscular dystrophy in mdx models [30] and patients [35] could aid therapeutic clinical management of patients affected of these disorders.

## Conclusions

Our study addresses the challenge of utilizing markers of energy metabolism to be used for translation in aiding the management of rare neuromuscular diseases patients. We demonstrate that the quantification of proteins of energy metabolism in a cohort of seventy three muscle biopsies of control donors and patients affected of different neuromuscular diseases offers sensitive and specific biomarkers that could be implemented to refine the understanding of the biological principles of rare diseases and, eventually, the management of these patients.

## Additional files

Additional file 1: Figure S1. Characterization of the antibodies produced. Representative Western blot analysis showing the reactivity of the different antibodies produced against recombinant proteins and native proteins in different human cell lines (HCT116, HepG2 and 293 T) and human liver. The antibodies (0.4 μg/ml) exclusively recognized the recombinant (R; 50–100 ng of protein) and native protein (truncated Aconitase I, AcoI; Citrate synthase, CS; glycerol-3-phosphate dehydrogenase 1, GPD1; lactate dehydrogenase A, LDH-A and NADH-ubiquinone oxidoreductase α-sub9, NADH-sub9). Note the lower electrophoretic mobility of the recombinant protein in most cases due to the tag used for purification. Additional file 2: Figure S2. Linear correlation between the fluorescence intensity and the content of protein in HCT116 cells. Cell extracts (0–1 μg/μl) were spotted in the arrays (see Figure 2A). Significant linear correlations were obtained between the fluorescence intensity (arbitrary units, a.u) of the spots and the amount the protein interrogated in the arrays. Protein concentrations in the biopsies were calculated by interpolation in the respective linear plots. Additional file 3: Figure S3. Validation of RPMA reproducibility. Histograms represent two different experiments (grey and red bars) of RPMA for GAPDH in different neuromuscular dystrophies/myopathies when compared to control donors, confirming that the results obtained with the RPMA approach are highly reproducible. Additional file 4: Figure S4. Quantitative analysis of the expression of proteins of metabolism represented as box plots. The Y axis indicates the values of intensity (a.u) calculated by interpolation in the linear plot of HCT116 cells and normalized by the expression values of β-actin. The X axis represents patient groups. Box plots represent the lowest, lower quartile, median, upper quartile, and highest observations of each marker in the different groups of pathologies. ○, outlier values and #, extreme values. * and **, p < 0.05 and p < 0.001 when compared to controls, respectively.

## Abbreviations

ACO1: Aconitase
α-F1-ATPase: α-subunit of the H^+^-ATP synthase
BMD: Becker Muscular Dystrophy
β-F1-ATPase: β-subunit of the H^+^-ATP synthase
COXI: Cytochrome c Oxidase subunit I
COXIV: Cytochrome c Oxidase subunit IV
CPTI: carnitine O-palmitoyl transferase I
CS: Citrate synthase
DMD: Duchenne Muscular Dystrophy
GAPDH: Glyceraldehyde-3-phosphate dehydrogenase
GPD1: glycerol-3-phosphate dehydrogenase 1
GlycV: Glycogenesis type V (Mc Ardle disease)
HADHA: Hydroxyacyl-CoA dehydrogenase
Hsp60: Heat Shock Protein 60 kDa
IF1: Inhibitor Factor 1 of the H^+^-ATP synthase
LDH-A: Lactate Dehydrogenase A
LGMD2C: Limb Girdle Muscular Dystrophy Type 2C
NADHs9: NADH dehydrogenase subunit 9
NCL: Neuronal Ceroid Lipofuscinosis
PDH: Pyruvate Dehydrogenase
PYGM: myophosphorylase
PK: Pyruvate Kinase
SDH: Succinate Dehydrogenase
SOD2: Superoxide Dismutase 2
Xp21 Carriers: symptomatic forms of DMD and BMD in female carriers
