# Supplementary figures and images for: Quantitative analysis of proteins of metabolism by reverse phase protein microarrays identifies potential biomarkers of rare neuromuscular diseases
Source: J Transl Med. 2015 Feb 18;13:65. doi: 10.1186/s12967-015-0424-1 (PMC4342896; doi:10.1186/s12967-015-0424-1)

## Slide 1
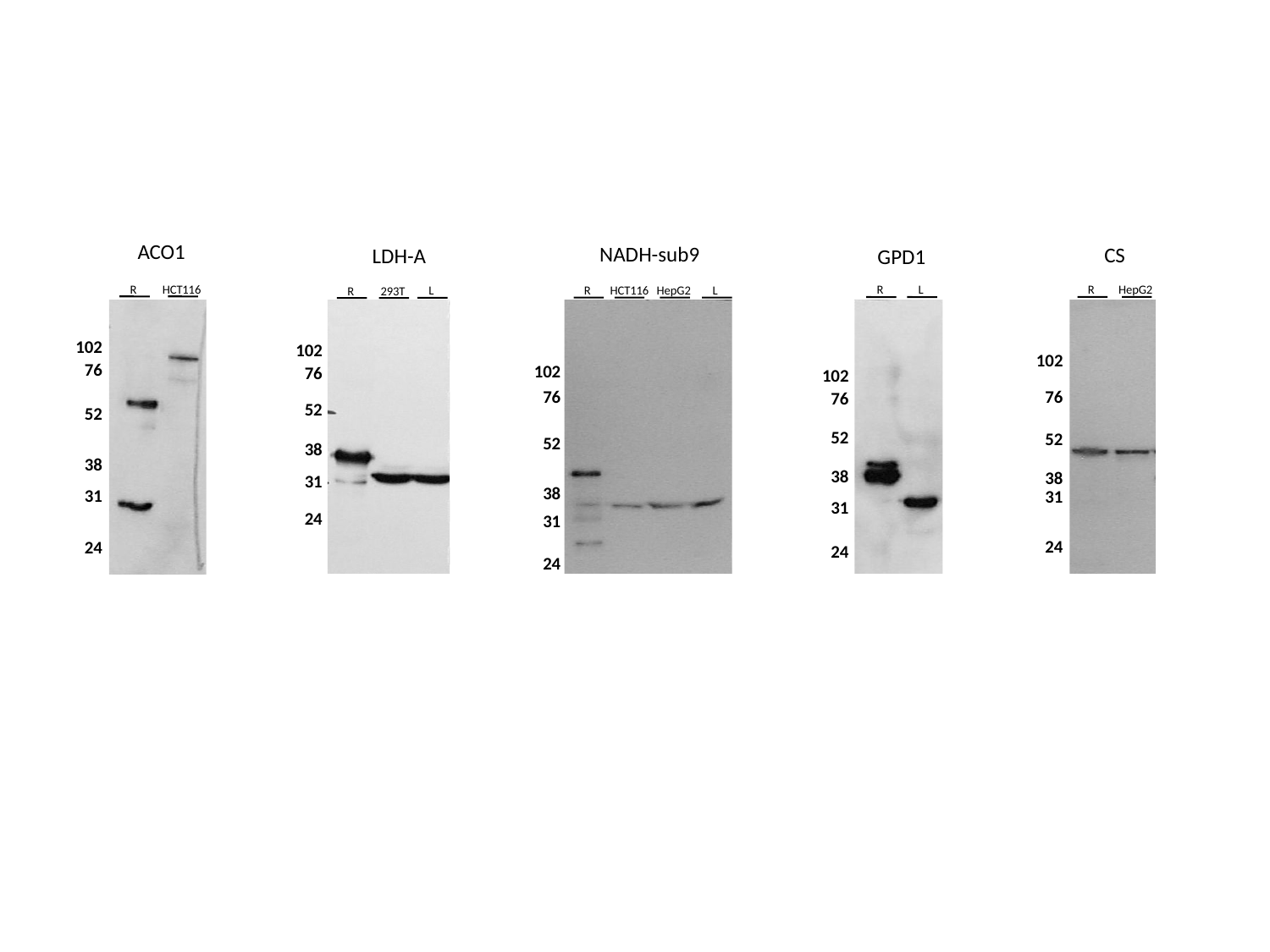

ACO1
NADH-sub9
 CS
 LDH-A
GPD1
 R
 HCT116
 R
 L
 R
 HepG2
 HepG2
L
 R
 HCT116
 L
 R
 293T
102
76
52
38
31
24
102
76
52
38
31
24
102
76
52
38
31
24
102
76
52
38
31
24
102
76
52
38
31
24

Supplement: Additional file 1: Figure S1. — Characterization of the antibodies produced. Representative Western blot analysis showing the reactivity of the different antibodies produced against recombinant proteins and native proteins in different human cell lines (HCT116, HepG2 and 293 T) and human liver. The antibodies (0.4 μg/ml) exclusively recognized the recombinant (R; 50–100 ng of protein) and native protein (truncated Aconitase I, AcoI; Citrate synthase, CS; glycerol-3-phosphate dehydrogenase 1, GPD1; lactate dehydrogenase A, LDH-A and NADH-ubiquinone oxidoreductase α-sub9, NADH-sub9). Note the lower electrophoretic mobility of the recombinant protein in most cases due to the tag used for purification. [file 12967_2015_424_MOESM1_ESM.ppt]

## Slide 1
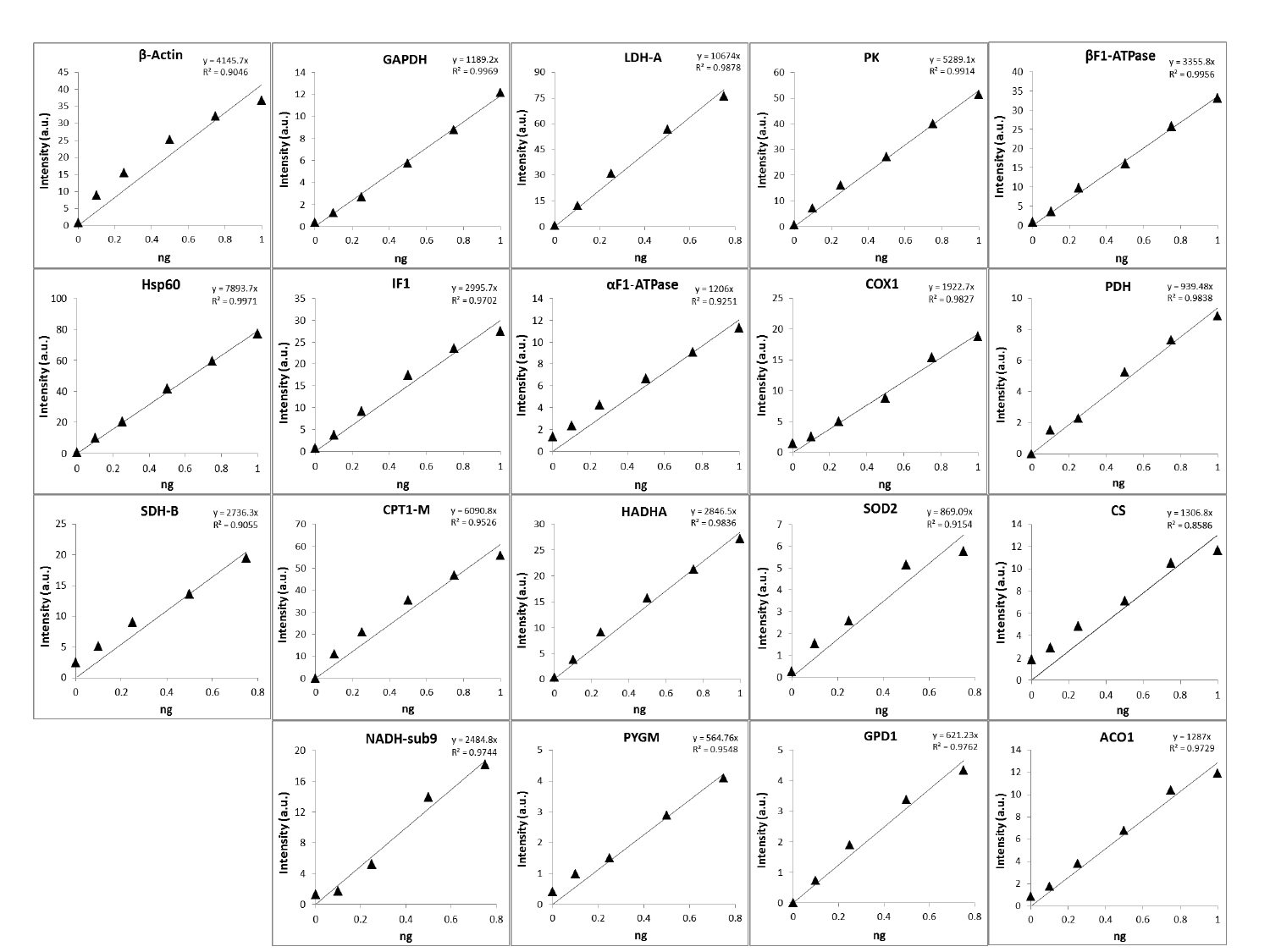

Supplement: Additional file 2: Figure S2. — Linear correlation between the fluorescence intensity and the content of protein in HCT116 cells. Cell extracts (0–1 μg/μl) were spotted in the arrays (see Figure 2A). Significant linear correlations were obtained between the fluorescence intensity (arbitrary units, a.u) of the spots and the amount the protein interrogated in the arrays. Protein concentrations in the biopsies were calculated by interpolation in the respective linear plots. [file 12967_2015_424_MOESM2_ESM.ppt]

## Slide 1
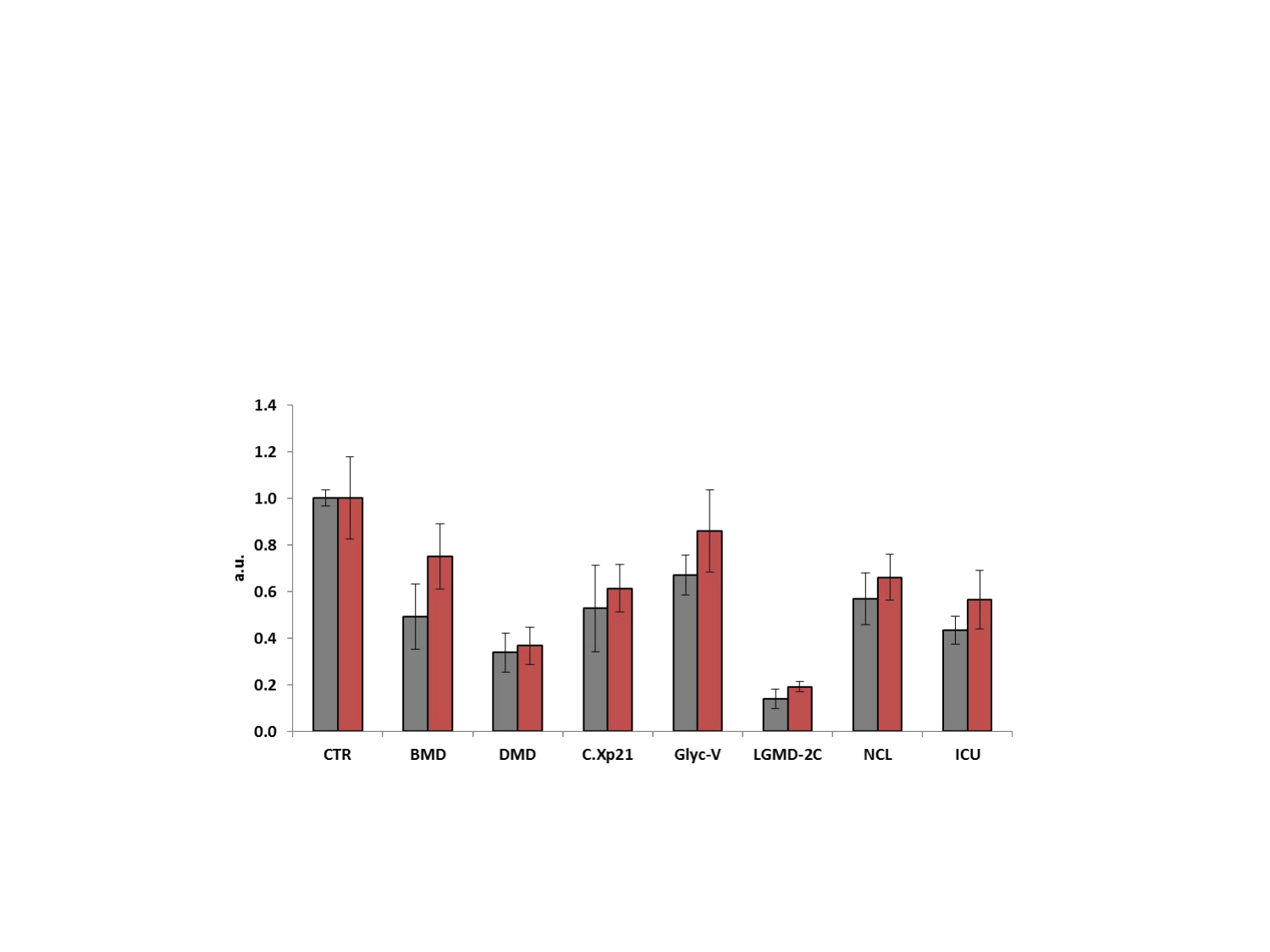

Supplement: Additional file 3: Figure S3. — Validation of RPMA reproducibility. Histograms represent two different experiments (grey and red bars) of RPMA for GAPDH in different neuromuscular dystrophies/myopathies when compared to control donors, confirming that the results obtained with the RPMA approach are highly reproducible. [file 12967_2015_424_MOESM3_ESM.ppt]

## Slide 1
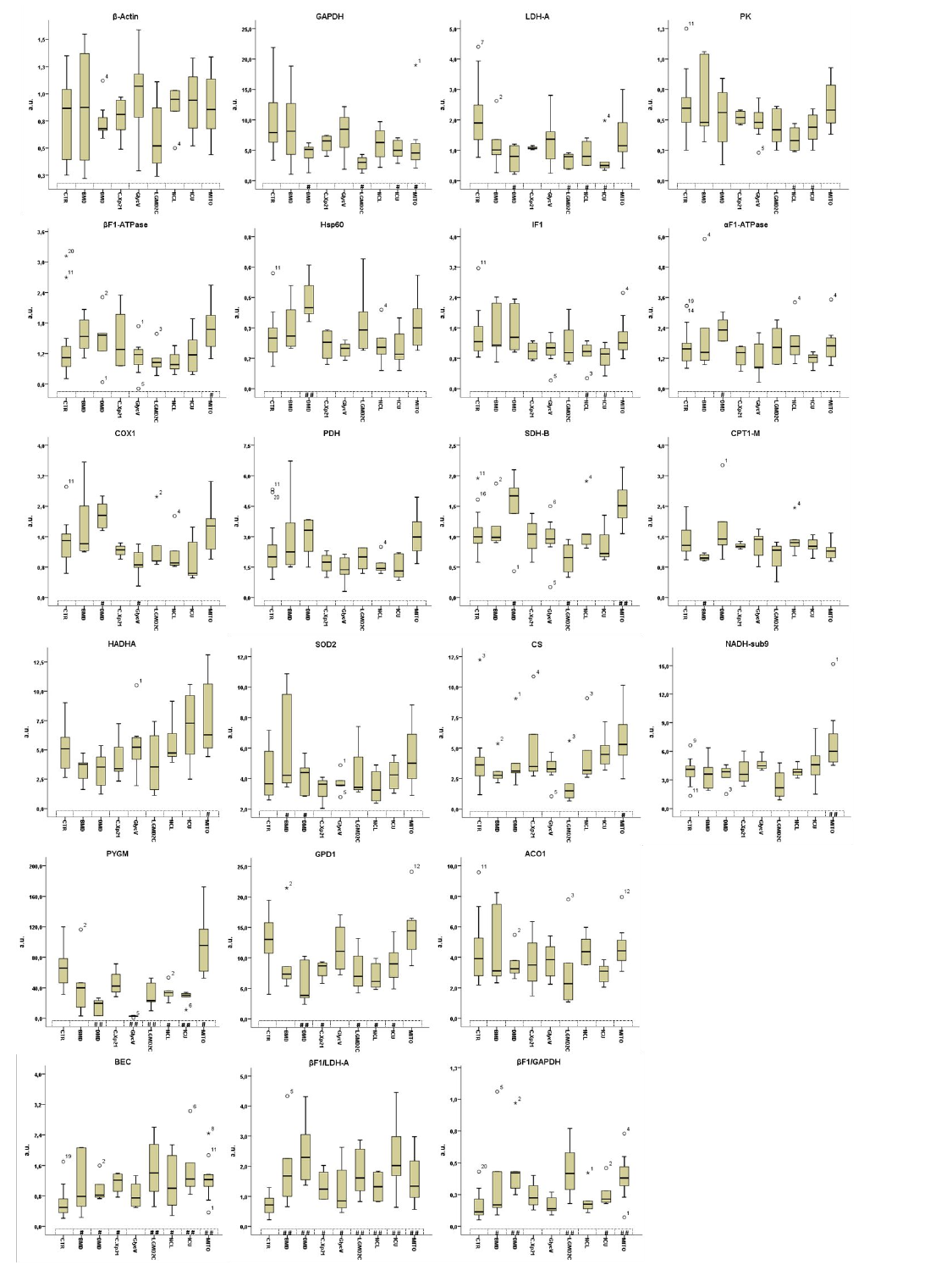

Supplement: Additional file 4: Figure S4. — Quantitative analysis of the expression of proteins of metabolism represented as box plots. The Y axis indicates the values of intensity (a.u) calculated by interpolation in the linear plot of HCT116 cells and normalized by the expression values of β-actin. The X axis represents patient groups. Box plots represent the lowest, lower quartile, median, upper quartile, and highest observations of each marker in the different groups of pathologies. ○, outlier values and #, extreme values. * and **, p < 0.05 and p < 0.001 when compared to controls, respectively. [file 12967_2015_424_MOESM4_ESM.ppt]
